# Commentary: Aortic valve structure: Entering the fourth dimension

**DOI:** 10.1016/j.xjtc.2021.08.041

**Published:** 2021-09-02

**Authors:** Shuab Omer, Faisal H. Cheema, Keshava Rajagopal

**Affiliations:** a Department of Clinical Sciences, University of Houston College of Medicine, Houston, Tex; b Houston Heart, HCA Houston Healthcare, Houston, Tex

**Figure:**
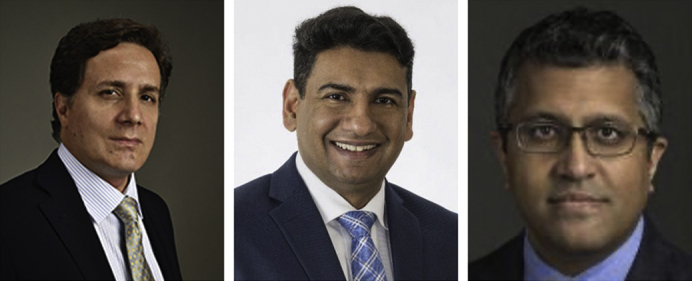
Shuab Omer, MD, Faisal H. Cheema, MD, and Keshava Rajagopal, MD, PhD

**Central Message**

Cardiac valvular function is assessed and understood in 4 dimensions—3 spatial, 1 temporal. Understanding cardiac valvular structure, as it causally impacts function, also requires this approach.

See Article page 200.

In this issue of *JTCVS Techniques*, Nijs and colleagues^1^ report a novel geometric methodology to assess bicuspid aortic valve (AV) structure. The authors studied cardiac-protocol computed tomographic angiography images in patients with bicuspid AV syndrome, over the range of Sievers classes of valve morphology. Examining “optimal” transverse (relative to the axial aortic centerline) circular cross-sectional images during diastole, the authors studied a variety of AV structural angles. Most important among these are: (1) α, the coaptation angle (formed by the nonfused commissures and the “coaptation point,” which corresponds to the luminal end of the raphe in a Sievers type 1 valve), and (2) β, the angle formed by the nonfused commissures and the center of the circular transverse cross-section of the AV. The central conclusion of the study is that α does not correlate well with commissural position, whereas β does. From this conclusion, the authors reasonably suggest that their findings have implications for the planning of AV repair operations. Thus, although the study is preliminary, it may be an important first step in understanding preoperative geometric and structural factors that influence AV reconstruction operative planning.

It is important to emphasize that α is more visually obvious, because the 3 points from which the angle is determined are based upon tissue landmarks: the 2 commissures, and the end of the raphe of the conjoint leaflet. In contrast, one of the points used to assess β does not correspond to a tissue landmark: the center of the circular cross-section. Thus, even though β may have an important role in assessing AV geometric structure, with implications for operative planning, its determination in the operating room may be challenging.

There are other even more important concerns aside from these technical ones, however. First, a single “best” circular transverse cross-section was used for analysis. However, and particularly importantly for the AV, each transverse cross-section within the aortic root has distinct structural features within it. Specifically, (1) the aortic annulus is not even a cylinder—rather, it is a stack of transverse conic sections with increasing diameter from proximal to distal; (2) related to this, leaflet geometry, and even structural architecture vary through these sections; and (3) as a consequence of (1) and (2) what coaptation actually means changes throughout space.

Second, the AV structure moves throughout the cardiac cycle. This motion will be unsteady (varying as a function of time), and particularly since the bicuspid AV is asymmetric, may be non-uniform (varying as a function of 3-dimensional space). However, with respect to AV competence, this being a diastolic property, the AV components ought not to be moving under normal circumstances. Yet, with respect to AV regurgitation, the diastolic aorta-to-left ventricle net force, regurgitant orifice “area” and resultant impedance to regurgitant flow, and regurgitant flow velocities, all may vary spatially and temporally. The angles α and β thus may vary through cross-sections (ie, through 3-dimensional space), and even through time. Thus, although this study is an important first step, far more work is required.

## References

[1] Nijs J., Vangelder B., Tanaka K., Gelsomino S., Van Loo I., La Meir M. (2021). Geometric characteristics of bicuspid aortic valves. J Thorac Cardiovasc Surg Tech.

